# The clinical diagnostic value of Xpert MTB/RIF for the detection of Mycobacterium tuberculosis in gastric aspirates

**DOI:** 10.1042/BSR20200138

**Published:** 2020-06-23

**Authors:** Hong-Kun Tan, Shu-Jin Fan, Yu-Cheng Xu, Jiong-Jiong Zhou, Yuan-Zhi Chen, Tian-Ao Xie, Zhi-Yong Pan, Yong Xia, Xu-Guang Guo

**Affiliations:** 1 Department of Clinical Medicine, the Third Clinical School of Guangzhou Medical University, Guangzhou 511436, China; 2 Department of Clinical Laboratory Medicine, the Third affiliated hospital of Guangzhou Medical University, Guangzhou, China; 3 Key Laboratory for Major Obstetric Diseases of Guangdong Province, the Third affiliated hospital of Guangzhou Medical University, Guangzhou 510150, China; 4 Key Laboratory of Reproduction and Genetics of Guangdong Higher Education Institutes, the Third affiliated hospital of Guangzhou Medical University, Guangzhou 510150, China

**Keywords:** Gastric-Aspirate, Mycobacterium-tuberculosis, Sensitivity, Specificity, Xpert MTB/RIF

## Abstract

**Background**: At present, the infection and prevalence rates of tuberculosis (TB) are still high in worldwide. The Xpert MTB/RIF technology has improved the diagnosis speed of Mycobacterium tuberculosis (MTB) and facilitated the rapid treatment of TB patients.

**Methods**: We searched experimental data derived from Xpert MTB/RIF for detecting MTB in gastric aspirates in PubMed, Embase, Web Of Science, and the Cochrane Library databases between January 2012 to April 2019. A summary receiver operating characteristic curve (SROC curve) was used to analyze the pooled sensitivity, pooled specificity, PLR, NLR, and DOR for determining the accuracy of the test.

**Results:** Our database search resulted in 10 relevant articles. The pooled sensitivity of Xpert MTB/RIF for detecting TB in GA was 86% (95% CI, 83–89%), and *I*^2^ = 93.4%. The pooled specificity was 92% (95% CI, 90–93%) and *I*^2^ = 97.8%. In addition, the positive LR was 12.12 (95% CI, 5.60–26.21), negative LR was 0.20 (95% CI, 0.11–0.36), and the diagnostic odds ratio (DOR) was 147.04 (95% CI, 37.20–581.19). Using the SROC curve, the AUC was 0.9730 and Q* was 0.9248 (SE = 0.0261). The publication bias was *P*=0.517 (*P*>0.05).

**Conclusions:** The Xpert MTB/RIF for detecting MTB in gastric aspirates was highly accurate. In addition, we observed that the publication bias in the present study was low. Hence, the Xpert MTB/RIF technology is highly accurate and has the advantage of rapid testing for MTB in clinical samples.

## Introduction

Tuberculosis (TB) remains a globally essential and leading cause of infectious disease. It was estimated that around 3 million TB patients were undiagnosed in 2013, with 10 million people diagnosed with TB in 2017 [1]. Approximately, 95% of TB infections and 99% of deaths due to TB infections occur in developing countries, with South Africa being the most affected country [2]. Hence, the early MTB diagnosis is very important. Sputum samples are the most commonly used material for diagnosis. Culturing MTB is the gold standard for TB diagnosis. However, it usually takes several weeks to produce the results needed for a proper diagnosis. Because the results cannot be quickly generated, timely diagnosis and patient treatment are delayed [3–5]. The WHO recommended method for the initial diagnosis of MTB is smear microscopy. However, due to the low sensitivity of microscopic examinations, the majority of patients will be misdiagnosed [6]. Hence, new methods are urgently needed to develop early diagnosis methods, shorten treatment times, and improve treatment efficacy and prevention.

In 2011, the MTB rapid molecular diagnostic Xpert MTB/RIF test was introduced. It has improved diagnostic sensitivity for detecting MTB [7]. At present, the Xpert MTB/RIF is used to diagnose bacterial infections in patients more accurately. Globally, the Xpert MTB/RIF test has reduced clinical misdiagnosis and has diminished the empirical treatment of patients with negative results. As previously demonstrated by Penz et al. [8], the Xpert MTB/RIF test has different sensitivities and specificities for detecting MTB obtained from lymphatic, pleural effusion, gastrointestinal tract, genitourinary system, cerebrospinal fluid, and other samples. Hence, the purpose of the present study was to perform a research from recently published studies that used the Xpert MTB/RIF test. The overall diagnostic accuracy for detecting MTB in gastric aspirates was then determined for its suitability for clinical diagnosis.

## Method

### Data sources and search strategy

The following search terms were used: Gastro Enteric, Gastrointestinal, Gastro-Intestinal, GI, Gastric Aspirate, GA, Xpert, Xpert MTB/RIF, Xpert RIF/MTB. The search strategy was performed independently by the authors using Pubmed, Embase, Web Of Science, and the Cochrane Library. Afterwards, the authors presented and compared their individual results. If they were inconsistencies, the search strategy was re-evaluated to resolve any differences. Through discussions, relevant studies were selected and used for the analysis.

### Study selection and data extraction

The inclusion and exclusion criteria are formulated first before reading the retrieved studies. The inclusion criteria as follow: (1) Each included study used Xpert MTB/RIF for detection of MTB and a standard test (culture). (2) Study assessing the accuracy of Xpert in gastric TB detection with reliable data to calculate true positive (TP), false positive (FP), false negative (FN), true negative (TN). (3) Humans samples are detected and analyzed. The exclusion criteria as follow: (1) duplicate studies, (2) animal experiments, (3) experiments of two groups of patients without contrasting and unrelated studies, (4) reviews, conference abstracts, case reports, and studies that cannot extract data.

Based on pre-established inclusion and exclusion criteria, the authors independently read the article title, abstract, keywords, etc. to determine whether the article was relevant. Afterwards, the authors compared their results. Any inconsistencies in article selection required all authors to read the full manuscript. The article in question was included or excluded after a consensus was reached through discussions.

The authors independently read the full article after the initial screening to determine whether the data were relevant and extractable. The extractable data included the name of the first author, publication year, country, sample size, gold standard, and true positive (TP), false positive (FP), false negative (FN), and true negative (TN) compared with Xpert and gold standard data. After data extraction, the authors collectively discussed the results and re-read articles with conflicting results. The authors sought additional advice from experts to resolve any differences. Afterwards, the extracted data were used to formulate the data feature table.

### Statistical analysis

Meta-disc (version 1.4) was used to analyze the data and determine the sensitivity, specificity, positive LR, negative LR, diagnostic OR, and SROC curve tables.

Each included study was quality assessed using Review Manager (RevMan V5.3, Cochrane Collaboration, Oxford, U.K.) software.

Publication bias was determined using STATA (version 12.0) at a test level of *α* = 0.05.

## Results

### Study identification and characteristics

**Figure 1:**
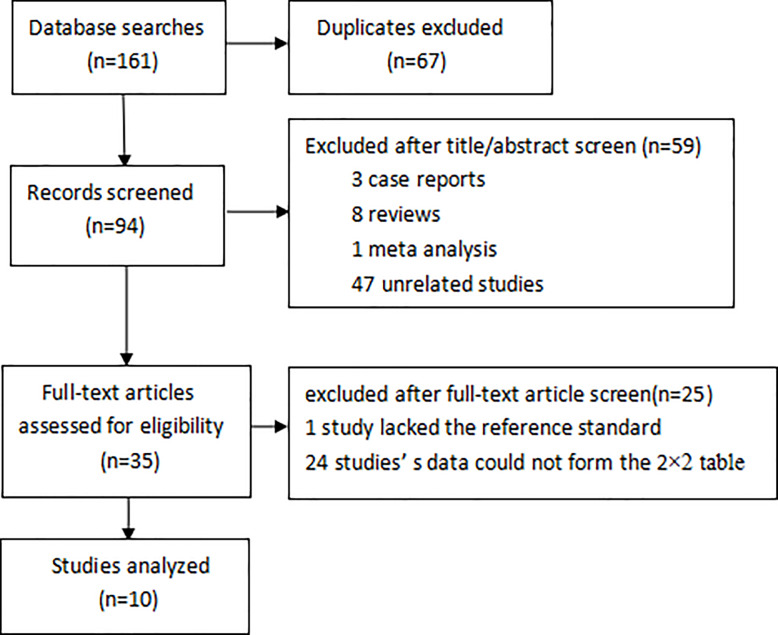
Flowchart of article retrieval

A total of 161 studies are retrieved: 48 studies from PubMed, 54 studies from WOS, 50 studies from Embase, 9 studies from the Cochrance Library, and 67 of the 161 studies are duplicates. The remaining 94 articles are excluded after reading the title and abstract of the article based on the inclusion and exclusion criteria, including 3 case reports, 8 reviews, 1 meta-analysis, and 47 unrelated studies. The remaining 35 articles exclude 25 articles after reading the full text, including 1 article lacking a gold standard comparison, and 24 articles cannot be used to form a 2 × 2 table for analysis based on their data. In the end, we got 10 articles for analysis (Figure 1).

The 10 selected articles [9–18] spanned from 2012 to 2018, of which 6 were published in Asia [9–14], 2 were published in Europe [15,16], and 2 were published in South Africa (Table 1) [17,18]. The number of study samples in each article ranged from 48 to 788, with a median of 245.5.

**Table 1:** Study characteristics of the selected publications

First Author	Year	Country	Specimen	Patients enrolled	Reference standard	TP	FP	FN	TN
Myo	2018	Myanmar	GA	231	culture	16	20	0	195
Hasan	2017	Pakistan	GA	48	culture	9	2	0	37
Aslam	2017	Pakistan	GA	267	culture	181	40	0	46
Lu	2017	China	GA	127	culture	25	40	0	62
Walters	2017	South Africa	GA	262	culture	18	3	15	226
Mazzola	2016	Italy	GA	356	culture	51	4	0	301
Singh	2015	India	GA	260	culture	52	19	25	164
Pang	2014	China	GA	211	culture	11	58	6	136
Bates	2013	Zambia	GA	788	culture	33	5	15	735
Tortoli	2012	Europe	GA	224	culture	48	0	10	166

### Study quality

**Figure 2:**
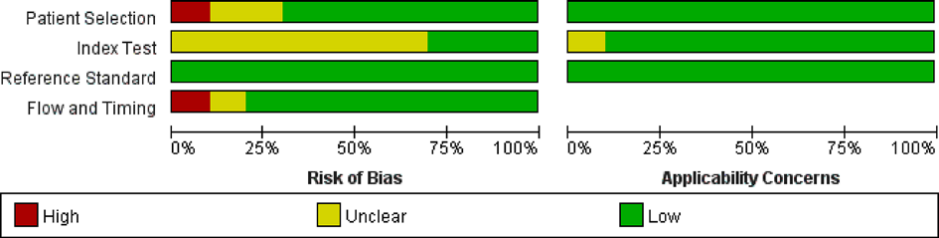
Risk of bias and applicability concerns graph for tuberculosis detection

The overall methodological quality evaluation of the Xpert MTB/RIF and bacterial cultures used in the study are summarized in Figure 2. The risk of bias due to patient selection, index testing, reference standards, procedures, and time were considered low.

### Sensitivity and specificity of Xpert MTB/RIF for diagnosis of MTB in GA

**Figure 3:**
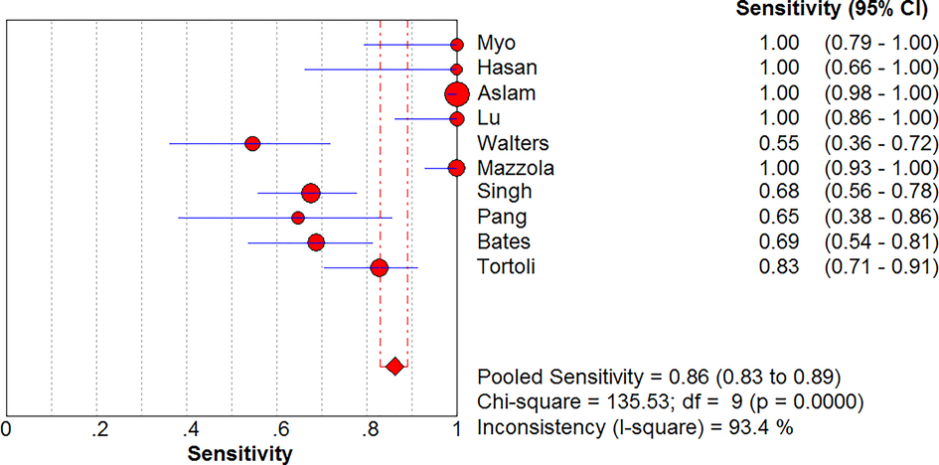
Forest plot of Xpert MTB/RIF sensitivity for tuberculosis detection in gastric aspirates

**Figure 4:**
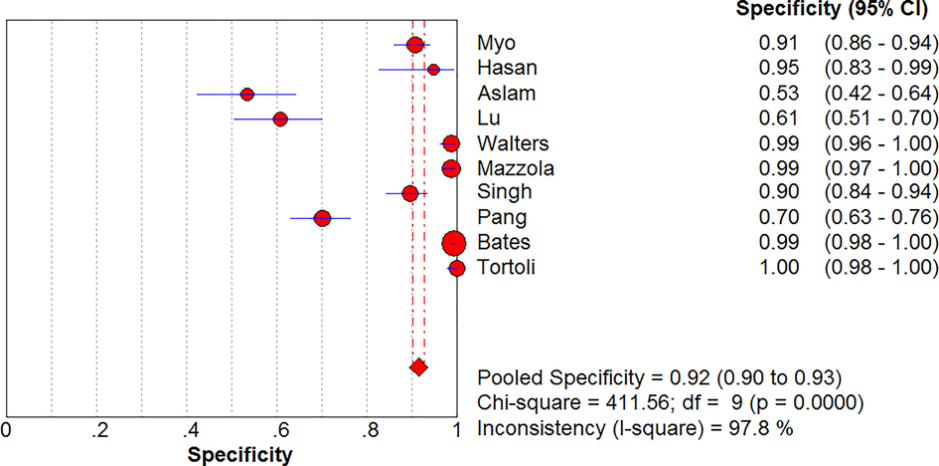
Forest plot of Xpert MTB/RIF specificity for tuberculosis detection in gastric aspirates

Ten studies included a comparison of the Xpert MTB/RIF versus cultures in 2774 GA samples for detecting tuberculosis (Figures 3 and 4). The sensitivity of Xpert MTB/RIF ranged from 55% (95% CI, 36–72%) to 100% (95% CI, 98–100%). The pooled sensitivity of Xpert MTB/RIF for MTB was 86% (95% CI, 83–89%), and the statistical value of *I*^2^ was 93.4%. The specificity of Xpert MTB/RIF ranged from 53% (95% CI, 42–64%) to 100% (95% CI, 98–100%). The pooled specificity of the Xpert MTB/RIF test for MTB was 92% (95% CI, 90–93%), and the statistical value of *I*^2^ was 97.8%.

### Combined positive LR and combined negative LR

**Figure 5:**
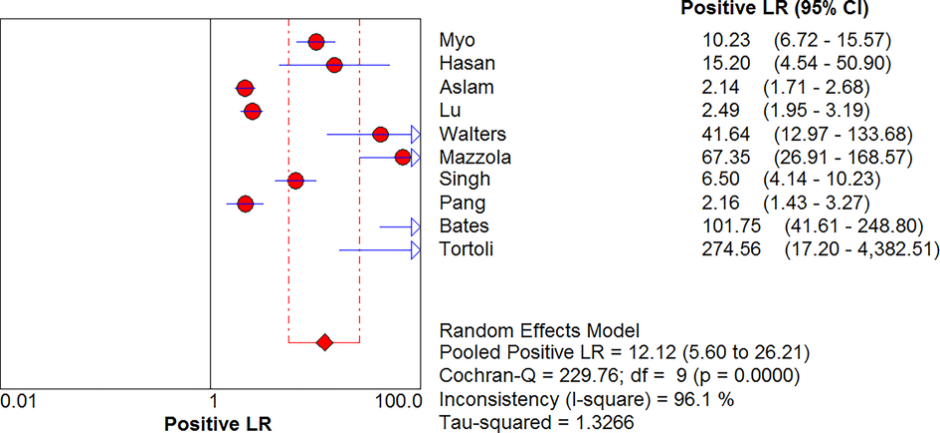
Forest plot of Xpert MTB/RIF positive LR for tuberculosis detection in gastric aspirates

**Figure 6:**
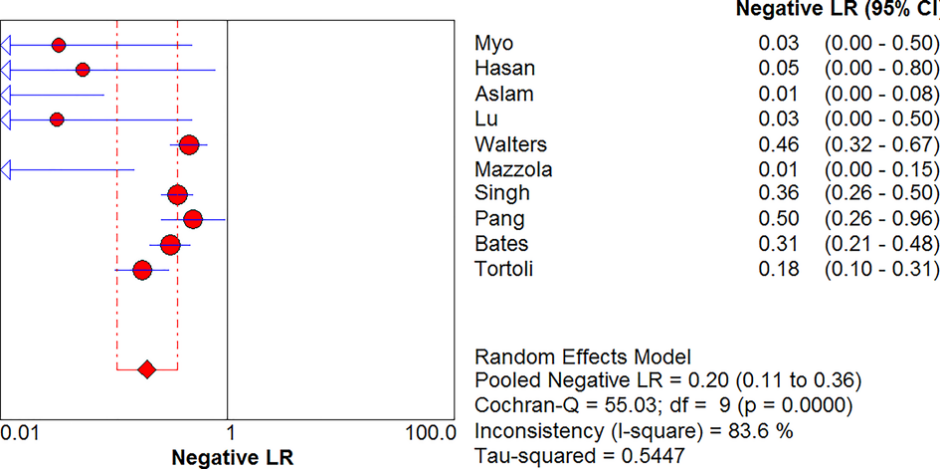
Forest plot of Xpert MTB/RIF negative LR for tuberculosis detection in gastric aspirates

**Figure 7:**
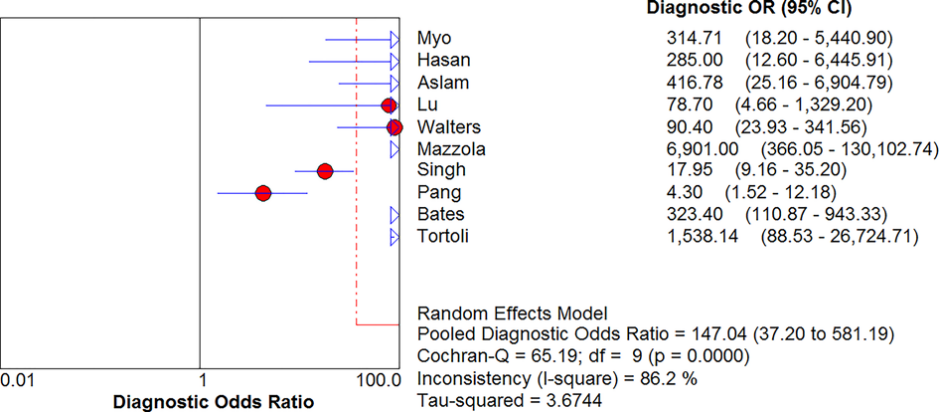
Forest plot of Xpert MTB/RIF diagnostic OR for tuberculosis detection in gastric aspirates

MTB in GA was detected using Xpert MTB/RIF, with a positive LR of 12.12 (95% CI, 5.60–26.21) and a negative LR of 0.20 (95% CI, 0.11–0.36) (Figures 5 and 6). The diagnostic odds ratio (DOR) was 147.04 (95% CI, 37.20–581.19) (Figure 7).

### SROC curve

**Figure 8:**
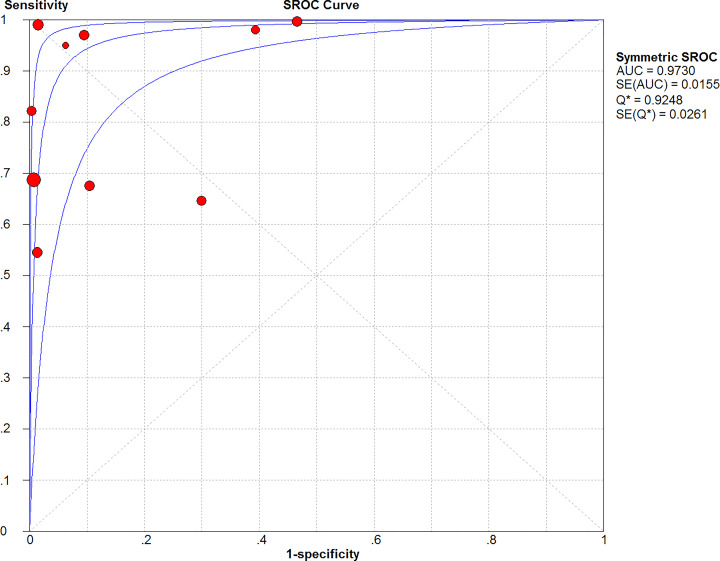
Summary receiver operating characteristic curves of Xpert MTB/RIF for tuberculosis detection in gastric aspirates

The AUC was 0.9730 determined from the SROC curve, indicating a good diagnostic value (Figure 8). In addition, Q* was 0.9248 (SE = 0.0261). The Q * value was close to 1, which indicated the high accuracy of Xpert MTB/RIF for MTB detection.

### Publication bias

**Figure 9:**
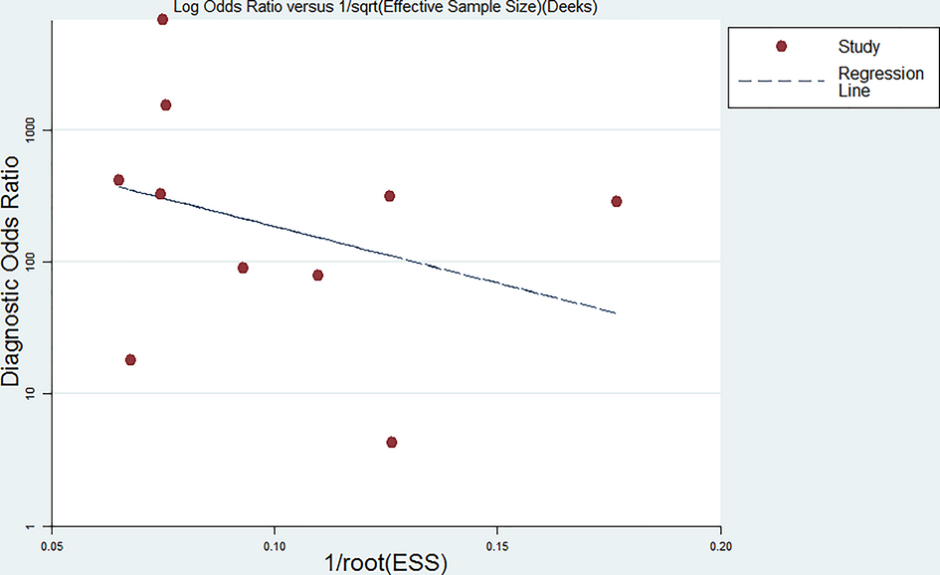
Deeks’ funnel plot indicating a low risk of publication bias (*P*=0.517)

The Deeks funnel plot was generated using the STATAS software. The *P*-value was 0.517 (*P*>0.05), indicating low publication bias (Figure 9).

## Discussion

In the present study, we extracted Xpert MTB/RIF data from 2774 GA samples obtained from 10 studies. The TB test was performed using GA samples, with bacterial cultures used as the standard. We found that the pooled sensitivity of the Xpert test was 86% (95% CI, 83–89%), and the pooled specificity was 92% (95% CI, 90–93%). In general, the Xpert MTB/RIF TB test performed using GA samples generated results that were relatively accurate compared with the culture method. The research performed by Penz et al. [8] in 2015, demonstrated that the Xpert MTB/RIF test had a sensitivity of 86% (95%CI, 67–98%), and specificity of 98% (95% CI, 98–100%) for analyzing GA samples. This was comparable to our results. However, their positive LR was 65 and negative LR = 0.18, which was different from our results (positive LR = 12.12). The reason may be that we included 10 studies for systematic analysis, while Penz el al. had only 5 studies in their research. In addition, the total sample size was much higher in our study. Hence, the use of the Xpert MTB/RIF test for detecting MTB in GA samples was highly accurate and could be used in clinical settings or public health systems to detect MTB infections in patients.

The standard diagnostic method for detecting MTB is through bacterial cultures [3–5]. We compared the Xpert MTB/RIF technology with the culture method. The Xpert MTB/RIF detects GA differently from the culture method. It is not affected by the acidity of the GA fluid, treatment delay of the bacteria, and excessive bacterial growth [9]. The diagnostic yield of Xpert MTB/RIF (31.4%) was significantly higher compared with the culture method (24.0%) [9]. Singh et al. [14] demonstrated that Xpert MTB / RIF could be used for diagnosing childhood TB even when the GA samples were stored for 4 years. Singh et al. also said in smear and culture-positive GA samples, the sensitivity of the Xpert MTB/RIF assay was 95.6% and was similar to the sensitivity reported for fresh clinical samples. Previous studies have demonstrated that Xpert MTB/RIF had a high detection rate and accuracy. We performed a rigorous search, using stringent screening criteria, and analyzed the data using strict statistical methods to improve the accuracy of our results.

Based on previous studies, the detection rate of MTB by microscopy was 48.94% and was prone to be affected by several factors [19,20]. The detection rate of Xpert MTB/RIF greatly exceeded the detection rate of microscopic examinations. Besides, the Xpert MTB/RIF test is convenient and rapid to perform.

Our work had a few limitations. The selected publications generated inconsistent data due to different instruments and technical protocols used. The low sensitivity and specificity caused from some of the studies had a significant influence on our research. Walters et al. [18] did not describe the technical details of processing their gastrointestinal fluid samples. Hence, we could not make a more objective evaluation of the low sensitivities they observed in their study. Aslams et al. [9] found that the specificity was only 53%. This could probably have been due to patients receiving anti-tuberculosis drugs. After treatment, some patients may have been cured. Hence there is a need to carefully evaluate the positive results from the Xpert MTB/RIF test [21]. Pang et al. [10] demonstrated relatively low levels of sensitivity and specificity. This may have been due to the long storage time of the samples (>3 days), and the MTB in the GA fluids being partially inactivated by the high acidic environment of the samples. It has been proven that MTB may be inactivated in an acid environment [22,23]. Their positive results from bacterial cultures were relatively low.

Additional studies using larger patient cohort samples should be performed to determine whether the Xpert MTB/RIF test could accurately detect MTB in GA samples.

The advantages of the Xpert MTB/RIF test are the reduced cost and faster turn-around times to generate results. This will prevent patients from being misdiagnosed and receive the appropriate treatment in a shorter period. Quicker and a more accurate diagnosis will lead to better patient prognosis, a better quality of life and reduced mortality.

## Abbreviations

DOR: diagnostic odds ratio
MTB: Mycobacterium tuberculosis
SROC: summary receiver operating characteristic
TB: tuberculosis
WHO: World Health Organization

## References

[1] Floyd K. et al. (2018) The global tuberculosis epidemic and progress in care, prevention, and research: an overview in year 3 of the End TB era. Lancet Respir. Med. 6, 299–314 10.1016/S2213-2600(18)30057-2 29595511

[2] Dye C. (2006) Global epidemiology of tuberculosis. Lancet 367, 938–940 10.1016/S0140-6736(06)68384-0 16546542

[3] Swaminathan S. and Rekha B. (2010) Pediatric tuberculosis: global overview and challenges. Clin. Infect. Dis. 50, S184–S194 10.1086/651490 20397947

[4] Cruz A.T. and Starke J.R. (2007) Clinical manifestations of tuberculosis in children. Paediatr. Respir. Rev. 8, 107–117 10.1016/j.prrv.2007.04.008 17574154

[5] Eamranond P. and Jaramillo E. (2001) Tuberculosis in children: reassessing the need for improved diagnosis in global control strategies. Int. J. Tuberc. Lung Dis. 5, 594–603 11467365

[6] Korenromp E.L. et al. (2012) Implementing the global plan to stop TB, 2011-2015–optimizing allocations and the Global Fund's contribution: a scenario projections study. PLoS ONE 7, e38816 10.1371/journal.pone.0038816 22719954 PMC3377722

[7] (2011) Policy Statement: Automated Real-Time Nucleic Acid Amplification Technology for Rapid and Simultaneous Detection of Tuberculosis and Rifampicin Resistance: Xpert MTB/RIF System. WHO Guidelines Approved by the Guidelines Review Committee, World Health Organization, Geneva 26158191

[8] Penz E. et al. (2015) Diagnostic accuracy of the Xpert® MTB/RIF assay for extra-pulmonary tuberculosis: a meta-analysis. Int. J. Tuberc. Lung Dis. 19, 278–284 10.5588/ijtld.14.0262 25686134

[9] Aslam W. et al. (2017) Gastric specimens for diagnosing tuberculosis in adults unable to expectorate in Rawalpindi, Pakistan. Public Health Action 7, 141–146 10.5588/pha.16.0126 28695088 PMC5493096

[10] Pang Y. et al. (2014) Evaluation of the Xpert MTB/RIF Assay in Gastric Lavage Aspirates for Diagnosis of Smear-negative Childhood Pulmonary Tuberculosis. Pediatr. Infect. Dis. J. 33, 1047–1051 10.1097/INF.0000000000000403 25361186

[11] Myo K. et al. (2018) Evaluation of Xpert((R)) MTB/RIF assay as a diagnostic test for pulmonary tuberculosis in children in Myanmar. Int. J. Tuberc. Lung Dis. 22, 1051–1055 10.5588/ijtld.18.0024 30092871

[12] Lu J. et al. (2017) The Feasibility of Xpert MTB/RIF Testing to Detect Rifampicin Resistance among Childhood Tuberculosis for Prevalence Surveys in Northern China. Biomed. Res. Int. 2017, 5857369 10.1155/2017/5857369 29359155 PMC5735616

[13] Hasan Z. et al. (2017) Evaluation of Xpert MTB/RIF testing for rapid diagnosis of childhood pulmonary tuberculosis in children by Xpert MTB/RIF testing of stool samples in a low resource setting. BMC Res. Notes 10, 473 10.1186/s13104-017-2806-3 28886729 PMC5591572

[14] Singh S. et al. (2015) Xpert MTB/RIF assay can be used on archived gastric aspirate and induced sputum samples for sensitive diagnosis of paediatric tuberculosis. BMC Microbiol. 15, 191 10.1186/s12866-015-0528-z 26420261 PMC4589030

[15] Mazzola E. et al. (2016) Performance of real-time PCR Xpert MTB/RIF in diagnosing extrapulmonary tuberculosis. Le Infezioni in Medicina: Rivista Periodica di Eziologia, Epidemiologia, Diagnostica, Clinica e Terapia Delle Patologie Infettive 24, 304–309 28011966

[16] Tortoli E. et al. (2012) Clinical validation of Xpert MTB/RIF for the diagnosis of extrapulmonary tuberculosis. Eur. Respir. J. 40, 442–447 10.1183/09031936.00176311 22241741

[17] Bates M. et al. (2013) Assessment of the Xpert MTB/RIF assay for diagnosis of tuberculosis with gastric lavage aspirates in children in sub-Saharan Africa: a prospective descriptive study. Lancet Infect. Dis. 13, 36–42 10.1016/S1473-3099(12)70245-1 23134697

[18] Walters E. et al. (2017) Xpert MTB/RIF on Stool Is Useful for the Rapid Diagnosis of Tuberculosis in Young Children With Severe Pulmonary Disease. Pediatr. Infect. Dis. J. 36, 837–843 10.1097/INF.0000000000001563 28151842 PMC5558052

[19] Steingart K.R. et al. (2006) Fluorescence versus conventional sputum smear microscopy for tuberculosis: a systematic review. Lancet Infect. Dis. 6, 570–581 10.1016/S1473-3099(06)70578-3 16931408

[20] Keflie T.S. and Ameni G. (2014) Microscopic examination and smear negative pulmonary tuberculosis in Ethiopia. Pan. Afr. Med. J. 19, 162 25810798 10.11604/pamj.2014.19.162.3658 PMC4362622

[21] Friedrich S.O. et al. (2013) Assessment of the sensitivity and specificity of Xpert MTB/RIF assay as an early sputum biomarker of response to tuberculosis treatment. Lancet Respir Med. 1, 462–470 10.1016/S2213-2600(13)70119-X 24429244

[22] Nhu N.T. et al. (2013) Evaluation of Xpert MTB/RIF and MODS assay for the diagnosis of pediatric tuberculosis. BMC Infect. Dis. 13, 31 10.1186/1471-2334-13-31 23343418 PMC3562258

[23] Parashar D. et al. (2013) Does neutralization of gastric aspirates from children with suspected intrathoracic tuberculosis affect mycobacterial yields on MGIT culture? J. Clin. Microbiol. 51, 1753–1756 10.1128/JCM.00202-13 23536406 PMC3716107

